# Marine Peptides as Potential Agents for the Management of Type 2 Diabetes Mellitus—A Prospect

**DOI:** 10.3390/md15040088

**Published:** 2017-03-23

**Authors:** En-Qin Xia, Shan-Shan Zhu, Min-Jing He, Fei Luo, Cheng-Zhan Fu, Tang-Bin Zou

**Affiliations:** Dongguan Key Laboratory of Environmental Medicine, School of Public Health, Guangdong Medical University, Dongguan 523808, China; enqinxia@163.com (E.-Q.X.); zss90y@163.com (S.-S.Z.); minjinghe0818@163.com (M.-J.H.); luofei_00@163.com (F.L.); fuchengzhan@126.com (C.-Z.F.)

**Keywords:** marine protein, bioactive peptide, regulation, glucose metabolism, structure active relationship

## Abstract

An increasing prevalence of diabetes is known as a main risk for human health in the last future worldwide. There is limited evidence on the potential management of type 2 diabetes mellitus using bioactive peptides from marine organisms, besides from milk and beans. We summarized here recent advances in our understanding of the regulation of glucose metabolism using bioactive peptides from natural proteins, including regulation of insulin-regulated glucose metabolism, such as protection and reparation of pancreatic β-cells, enhancing glucose-stimulated insulin secretion and influencing the sensitivity of insulin and the signaling pathways, and inhibition of bioactive peptides to dipeptidyl peptidase IV, α-amylase and α-glucosidase activities. The present paper tried to understand the underlying mechanism involved and the structure characteristics of bioactive peptides responsible for its antidiabetic activities to prospect the utilization of rich marine organism proteins.

## 1. Introduction

Diabetes mellitus is known as a seriously chronic metabolism disorder. According to the forecast, the prevalence of type 2 diabetes mellitus (T2DM) will increase from 350 million today to 592 million by 2035 [1]. The nutrient overload in prolonged periods was the key factor causing the bad situation due to impairing the pancreatic β cell function. In sequence, insulin resistance, the impaired secretary insulin and glucose tolerance become true, and the following result is to develop T2DM [2,3,4]. Fortunately, recent studies of Zhu et al. suggest that peptides and protein hydrolysates from wild *Chum Salmon* (*Oncorhynchus kern*) skin markedly decreased the level of fasting glucose level and the pancreatic apoptosis of islet cells [5]. Pandey et al. reported that the bacteria associated with marine sponge, *Aka coralliphaga*, produce many glucosidase inhibitory peptides [6]. Among marine microalgae, *Chlorella vulgaris* (*C. vulgaris*) is regarded as a complementary medicine, due to its supplements, and exhibited benefits for some health disorders, such as dyslipidemia, hyperglycemia, and hypertension as well as weight loss in several studies [7,8]. These results indicated that the natural marine bioactive peptides can improve the deleterious process of T2DM. However, few reports involved antidiabetic activities of marine natural peptides can be found in literature in our knowledge. Therefore, the present review will summarize all evidence on antidiabetic activities of natural peptides from milk, bean and marine organisms. Its primary coverage involved the response of insulin-regulated glucose metabolism and dipeptidyl peptidase IV (DPP-IV), α-amylase and α-glucosidase activities on the bioactive peptides. The mechanism underlying each antidiabetic activity and the structure characteristics of bioactive peptides responsible for its antidiabetic activities were also carefully discussed for the future novel marine peptides investigation.

## 2. Regulation of Bioactive Peptides on the Insulin-Regulated Glucose Metabolism

### 2.1. Protecting Pancreatic β-Cells of Bioactive Peptides

Adequate insulin secretion is necessary to maintain blood glucose levels within a physiological range, and competent pancreatic β-cells are responsible for that task. However, it becomes insufficient for the T2DM individuals due to their pancreatic β-cell failure [9]. The major factor causing the adverse effect on pancreatic β cells was reported as chronic nutrient overload, which causes a cell to increase its function and mass to match the increasing nutrient availability and insulin resistance [10]. As it can not adapt to maintaining glucose homeostasis at the high glucose challenges over prolonged periods [4], hyperglycemia was developed, which was verified to generate over-production of free radical species including reactive oxygen species (ROS) and nitric oxide (NO) radicals. These free radicals can cause defects in the mitochondrial respiratory chain by excessive requirements of oxidative enzymes activities (e.g., NOX), protein glycation and non-enzymatic oxidation and peroxidation of carbohydrates and lipids, and so on. Ultimately, increasing oxygen free radicals generate in various tissues including pancreas and kidneys [11,12,13]. Based on in vitro cell models and animal models of diabetes assays, researchers found that increased intracellular ROS production can significantly promote peripheral insulin resistance and induce endoplasmic reticulum (ER) stress, mitochondrial dysfunction, apoptosis and cell death. Subsequently, pancreatic β cell mass become deficient and its secretory function was impaired [14,15,16,17,18,19,20]. Using Min6 cells and pancreatic islets, Mailloux and coworkers found that an increase in mitochondrial matrix ROS can reverse the glutathionylation of uncoupling protein-2, which subsequently impedes glucose-stimulated insulin secretion from β cells [21]. In addition, it is reported that insulin-secreting β cells of the pancreatic islets contain gene expression and activity of the H_2_O_2_-reducing enzymes catalase and glutathione peroxidase (GPx) in islets accounting for only 1%–5% of the values in liver, and levels of cytosolic and mitochondrial superoxide dismutases (SOD) in islets only around 30% of those in the liver [22,23]. Obviously, as induced by chronic hyperglycemia and exposure to proinflammatory cytokines, the poor antioxidant defense capability of pancreatic β cells was sensitive towards oxidative stress [23,24,25]. Overexpression of these antioxidant enzymes has been observed to protect various β cell lines against oxidative damage [26,27,28]. Reactive oxygen species sensitized by metallothionein and catalase in nonobese diabetic mice was predicted to protect the pancreatic β cells from autoimmune destruction in male non-obese diabetic (NOD) [29]. Therefore, control of oxidative stress and inflammatory may be key approaches to reduce pancreatic β cell damage and the development of T2DM [30].

Recently, researchers reported that natural nutrient metabolites can exert a significant role to preserve β cell functions and mass, prolong the pre-diabetic phase and delay the progress to frank T2DM. For instance, consumption of dairy products was found linking with a decrease in the risk of type 2 diabetes [31,32]. Nasri et al. also reported that orally administered goby fish protein hydrolysates, not undigested goby fish protein, can significantly attenuate hyperglycemia and restored the antioxidant status under high-fat-high-fructose diet-induced oxidative stress in rats [33]. The same results reported that the effectiveness of the natural administration of fish protein hydrolysates, produced from *Sardinella aurita* and *Salaria basilisca*, in improving the oxidative status antioxidant for cholesterol-fed rats and alloxan-induced diabetic rats, respectively [34,35]. The results suggest that the presence of potent active peptides in fish protein hydrolysates was effective in enhancement of the antioxidant status.

Using in vitro assays, scavenging free radical capacity of bioactive peptides was observed by several researchers [14,36,37,38]. After being treated with pinto Durango bean (*P. vulgaris* l.) alcalase hydrolysates for 20 h at the concentration of 100 μg/mL, ROS production due to *tert*butyl hydroperoxide (t-BOOH) was almost eradicated in in vitro cell assay [36]. Fernández-Tomé [37] also found that lunasin, a soy peptide, exerted an effective scavenger as high as 190% of the intracellular ROS generation in HepG2 cells due to exposed to t-BOOH, compared to the control. In addition, the receptor in HepG2 cells for advanced glycation end products (RAGE) showed the lowest expression treated with the complete protein hydrolysates. While RAGE was found in pancreatic islets acting as an inducer of pancreatic β-cell apoptosis and developing of chronic diabetic complications via nicotinamide adenine dinucleotide phosphate oxidase mediated ROS generation in vitro assays [38]. Treatment with β-casomorphin-7, a milk-derived bioactive peptide, a considerable reduction in H_2_O_2_ content (*p* < 0.05) and a remarkable increase in the activity of GSH-peroxidase, SOD and catalase of the anti-oxidation system were observed. Simultaneously, the abatement of free-radical-mediated oxidative stress in blood and myocardium and cardiac indexes were also observed [39]. Protective effect of peptides on pancreatic β-cells against intracellular ROS due to a high glucose exposure has also been observed [14].

Natural peptides were also reported to efficiently ameliorate the diabetes symptoms. The levels of blood glucose of streptozotocin-induced diabetic rats markedly decreased after treatment with β-casomorphin-7, compared with model control group (*p* < 0.01) [39]. Bioactive peptides were observed to reduce the expression of cytokines such as interleukin-1β and tumor necrosis factor-α in pancreatic β-cells, which both generate as the cells were exposed to high glucose in vitro [40]. A Chlorella-11 peptide was also able to suppress lipopolysaccharide-induced nitric oxide (NO), serum TNF-α and inflammation [41]. In addition, it was reported that the common bean peptides can upregulated the expression of insulinlike growth factor 2 (IGF-II), a kind of adipokines in pancreatic β-cells now being believed to play a negative role in the development of obesity-associated insulin resistance and anti-inflammation [42].

### 2.2. Enhancement of Glucose-Stimulated Insulin Secretion

It has been revealed that T2DM develops when the insulin secretory capacity is unable to compensate for the increase of insulin resistance. The incretins, gut-derived hormones released from small intestine enteroendocrine cells (EECs), i.e., glucagonlike peptide 1 (GLP-1) and glucose dependent insulinotropic peptide (GIP), exert the significant role in regulation of food digestion by stimulation of glucose-dependent insulin secretion, as well food intake by promoting satiety to decrease appetite [43,44,45]. However, studies showed that circulating GLP-1 levels increase after meal intake but rapidly decrease 80%–90% due to cleaved by dipeptidyl peptidase IV (DDP-IV) [46]. Therefore, the DPP-IV inhibitors have indirect effects on islet function via contributing to insulin secretion and lowering blood glucose by increasing incretin action [47]. As early as 1988, Liddle et al. found that protein digestion can stimulate gut hormone secretion and expression in rats [48]. According to Caron et al., intestinal digestion derived from bovine haemoglobin exhibited significant efficiency on gut hormone release and DPP-IV activity inhibition, and those hormones’ gene expression was also up-expressed [49]. The DPP-IV inhibition capacity of some diet origin peptides above 200 μM of in literature is displayed in Table 1.

From Table 1, milk is the main source of peptides with efficient DPP-IV inhibitors in literature. Skin from halibut, tilapia and deer also showed significant DPP-IV inhibition capacity with IC 50 lower than 200 μM. Plant proteins digested in vitro or in vivo have been investigated the DPP-IV inhibitory peptides by some researchers, such as cowpea bean [67], Quinoa [68], rice bran [69], raw amaranth flour, soybean flour, and wheat flour [70]. However, except for Macroalga *Palmaria palmate*, DPP-IV inhibition capacity were exhibited with IC 50 far higher than 200 μM. The collected data showed that novel original peptides from natural proteins, especially from marine organisms, have been widely investigated for the management of T2DM.

### 2.3. Regulation of Glucose Uptake and Lipid Accumulation

Hyperglycemia has been identified as a key factor to induce to deficiency in insulin secretion and/or decreased reaction of the organs to insulin (World Health Organization, 1999). Besides diet and lifestyle modifications, control and prevention of hyperglycemia are primary approaches in the management of diabetes mellitus, which is involved in several physiological processes, such as increasing utilization of the glucose by the peripheral tissues and lowering hepatic glucose output and adipocyte fat-accumulation [71].

Some studies showed that natural peptides can correct high blood sugar, even without regulation of insulin secretion. For instance, the bean hydrolysates from pinto Durango had a dose-dependent insulin sensitizing effect (*p* < 0.05) comparing to the control. The most potent fraction was pinto Durango-alcalase < 1 kDa, which caused insulin resistant cells to increase (67 ± 3.2)% of glucose uptake compared to the non-insulin resistant cells [37]. The plasma glucose was also significantly decreased (25%–34%), after simultaneously intervening rats high-fat-high-fructose diet (HFFD) and goby fish protein hydrolysates, compared to the HFFD group [33]. β-casomorphin-7, a peptide from milk, was also found to restrain the elevation of blood glucose, and its effect is slightly inferior to insulin (11.18 ± 0.72 to 14.92 ± 0.66 mmol/L) [39]. The same results were found that the hypoglycemic effect of protein hydrolysates from muscle fish *Zebra blenny* in alloxan-induced diabetic rats [35] and from the vegetable *Momordica charantia* L. in alloxan-induced diabetic mice [33]. Yuh et al. found significantly enhanced hypoglycemic effects of *chlorella* consumption on streptozocin (STZ) induced diabetic mice [72]. By the similar assays, Jeong et al. observed significant improvement of insulin sensitivity in type 2 diabetic and normal Wistar rats., but the glucose-stimulated insulin secretion had not been influenced by chlorella consumption [73] Aglycin, a peptide from soy, exhibited effectively in preventing hyperglycemia in a diabetic animal model with impaired glucose tolerance and insulin resistance, which were induced in BALB/c mice (i.e., the laboratory bred strain of albino mice specially used for the study of cancer, neurological diseases) with a high-fat diet and received a single intraperitoneal injection of STZ [74]. Aglycin reduced blood glucose levels by 45.0% after long-term treatment (aglycin vs. model day 21 7.3 ± 0.5 vs. 11.3 ± 0.4 mmol/L; day 28 7.1 ± 0.2 vs. 12.4 ± 0.6 mmol/L, Pb.01, respectively) [74]. The notable efficiency has also been observed by Veloso et al. [75]. However, insulin secretion and body weight control in aglycin treated mice was not affected. Glucose levels were lowered after insulin loading in aglycin-treated mice in the insulin tolerance test. It indicated that glucose control induced by aglycin is largely mediated by enhancing glucose utilization and insulin sensitivity in peripheral insulin target sites [74]. Peptides from salmon hydrolysate, separated by electrodialysis with filtration membrane, also enhanced glucose uptake in L6 skeletal muscle cells by up to 40% without insulin increase [76].

Recently, researchers found that there was an inverse relationship between amount of secretory adiponectin, known as an insulin sensitizor, and the percentage of adipose tissue in the internal organs. It indicates that the decreased fat accumulation may improve glucose tolerance by the enhancement of insulin sensitivity [77]. According to results of Toledo et al., lipid accumulation was inhibited from 13% to 28% when adipocytes were treated with the bean hydrolysates throughout the differentiation process, and their <1 kDa peptide fraction showed slightly higher than whole hydrolysates [36]. Similarly, Martinez-Villaluenga et al. showed an inhibition of lipid accumulation from 27% to 46% in 3T3-L1 adipocytes when treated with alcalase soy hydrolysates at a concentration of 100 μM (1000 μg/mL) with an average of molecular mass of 10 kDa [78]. This study showed that common bean hydrolysates have the inhibitory effect on lipid accumulation. Lipid accumulation in adipose tissue can be reduced by different mechanisms, e.g., reducing lipid uptake through suppressing lipoprotein lipase or reducing lipid synthesis through inhibiting fatty acid synthase (FAS) [79]. To investigate the effect of peptides from natural protein on liquid accumulation, human white pre-adipocytes (HWP) received intervention from 11 peptides from fish, seaweed, shellfish, in order to investigate proliferation, differentiation and maturation. The results showed that Ala-Pro, Val-Ala-Pro and Ala-Lys-Lys greatly affected viability of HWP during the proliferation period, while Lys-Trp and Val-Trp reduced the number of viable cells during the differentiation stage. The decrease of their final lipid content and of the mRNA level of adipocyte markers (aP2, GLUT4, LPL and AGT) was also involved. Kim et al. showed that the peptide GAGVGY also downregulates lipid accumulation modulating of gene expression such as sterol regulatory element-binding proteins-1c, Peroxisome proliferator-activated receptor gamma and fatty acid synthase [80], which exhibited a dual effect regulating glucose uptake and lipid accumulation in a similar way as the present results [81,82,83]. In literature, some peptides from goat and soybean have been reported to inhibit growth of preadipocytes, decrease the differentiation process and decrease the final lipid content in human white preadipocytes and lipid accumulation [79,81]. These results suggest that natural peptides may be a potential compound on regulation of T2DM via inhibitory lipid accumulation.

### 2.4. Regulation of the Insulin-Signaling Pathways

**Figure 1:**
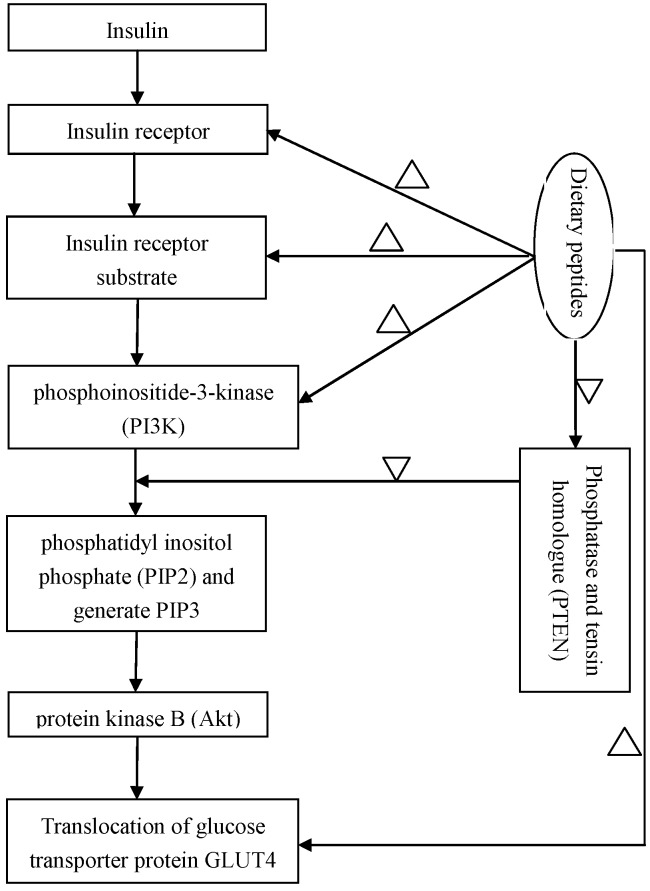
Some regulation evidence of natural peptides on the insulin-signaling pathways. Note: ‘∆’ and ‘▽’ mean the natural peptides display upregulation and downregulation on the corresponding bioprecessed, respectively.

Correction insulin resistance is an important therapeutic strategy for T2DM. Insulin resistance is a physiological condition, in which cells fail to respond to the normal actions of the hormone insulin, and reduction or impairment of insulin-stimulated glucose uptake. Insulin receptor substrate-1/phosphoinositide-3-kinase/protein kinase B (IRS-1/PI3K/Akt) signaling pathways was found as a main target to correct insulin regulating glucose uptake [84]. A defect in protein kinase B (PKB or Akt) signaling that reduces the translocation of the glucose transporter protein GLUT4 to the cellular membrane may be a main impairment of insulin-stimulated glucose uptake under insulin resistance conditions [85]. The action mechanism of IRS-1/PI3K/Akt signaling pathways on regulation of blood sugar is illustrated in Figure 1. In vivo assays showed that under insulin resistance of diabetes, phosphorylation level of Akt Ser473 decreases, and insulin signal transduction also significantly abates due to a decrease in insulin receptor concentration and kinase activity [86,87]. Therefore, insulin receptor (IR), insulin receptor substrate-1/2 (IRS-1/2), PI3K and Akt all might be efficient targets to regulate downstream signaling cascade to lower blood sugar level.

Some evidence has reported on the upregulation of GLUT4 in T2DM individuals by natural peptides. The effect of aglycin, a peptide from soy, on insulin signaling in the mice skeletal muscle showed that a significant increase in the expression of IR and IRS1 genes, as well as total IR, IRS1, p-Akt protein and membrane GLUT4, was observed. An increase of 75% of basal glucose uptake was found in both normal and insulin-resistant C2C12 cells [74]. Β-casomorphin-7 and insulin increased (*p* < 0.05) 1.37-fold and 1.62-fold of the expression of GLUT-4 in myocardium, respectively. In contrast with the model group, soybean peptides also have been reported to improve insulin action via increasing the expressions of GLUT4 and insulin regulatory genes in diabetic animals [88,89]. The peptide GAGVGY, a fibroin derivative, increased both basal and insulin stimulated glucose uptake through enhancement of GLUT1 expression and PI3K-dependent GLUT4 translocation [80]. Tripeptides such as GEY and GYG, derived from the peptide E5K6 from silk, stimulated glucose uptake in 3T3-L1 adipocytes by inducing the expression of glucose transporters GLUT1 and GLUT4 [90].

In adipose tissue, insulin is also responsible for the enhanced uptake of glucose by GLUT4. When adipocytes show insulin resistance, GLUT4 does not translocate to cell membrane in response to insulin release by the pancreas, and this leads to reduced glucose uptake. Common bean peptides modulating glucose transporters in insulin resistant adipocyte 3T3-L1 were evaluated by confocal microscopy. Results showed that there was a dose-dependence between the upregulation expression of glucose transporters GLUT4 and fraction peptides with <1 kDa derived from alcalase and bromelain [36]. In addition, a fermented soybean extract significantly increased the expression of GLUT4 and glucose uptake in 3T3-L1 adipocytes [91].

In vivo assays showed that fat accumulation was strongly associated with the inhibition of the PI3k signaling pathway, which was involved in the inhibition of insulin signaling [92]. Recovery of the activity of PI3K/Akt could protect the liver from non-alcoholic fatty liver disease induced injury [93]. These results might explain the fact that long-term feeding of soy peptide induced weight loss in obese mice both in healthy and diabetic animal models [74,94].

In addition, Akt is one of the major downstream targets of PI3K and responsible for the physiological function of insulin in adipocytes [95]. Phosphatase and tensin homologue (PTEN) is a lipid phosphatase that downregulates the action of PI3K decreasing insulin signaling, playing a role in regulating glucose metabolism [96]. A reduction of PTEN was presented with pinto Durango-bromelain bean hydrolysate and its <1 kDa peptide fraction treatments [36].

### 2.5. Clinical Trials

In very rare clinical human studies on anti-diabetes peptides, the efficacy of *Chlorella vulgaris* (*C.* *Vulgaris*) in prevention and treatment of dyslipidemia, hyperglycemia, hypertension as well as weight loss was found in literature [97,98,99]. *C. vulgaris*, a single-celled marine green algae, has been regarded as a complementary medicine [100]. Studies showed consumption of chlorella tablets for 16 weeks led to ameliorate insulin signaling pathways and noticeable reduction in serum glucose concentrations [97]. Panahi et al. also reported that *C.* *vulgaris* supplementation results in a marked decrease in insulin resistance and fasting serum glucose level in non-alcoholic fatty liver disease (NAFLD) patients [98]. Recently, Ebrahimi-Mameghani et al. recruited 70 obese patients with NAFLD aged 20–50 years to interfere with *C. vulgaris* supplementation. The results showed that 1200 mg *C. vulgaris* supplementation brought several potential beneficial effects, such as loss of weight, lowering serum glucose level, improvement of inflammatory biomarkers and liver function in NAFLD patients [99].

## 3. Inhibition of Bioactive Peptides to α-Amylase and α-Glucosidase Activities

Other approaches to decrease hyperglycemia are to control or delay glucose absorption by inhibition of α-glucosidase or α-amylase in the gastrointestinal tract. α-amylase is an enzyme that hydrolyses α-bonds polysaccharide such as glycogen and starch to oligosaccharides. α-Glucosidase is present in the epithelial mucosa of the small intestine and cleaves glycosidic bonds in oligosaccharides, releasing monosaccharides into blood sugar [30,101]. Thus, inhibition of α-amylase and α-glucosidase is an alternative pathway to management of the blood glucose levels and T2DM [102].

A significant increase in the serum α-amylase activity (by 86.08%) was found in HFFD-fed rats compared to control rats (*p* < 0.05). As oral administration of goby fish protein hydrolysates, the α-amylase activity in that of high-fat-high-fructose feed rats decreased by about 62% compared to the HFFD group [35]. Pinto bean peptides (<3 kDa fraction) and cumin seed-derived peptides also showed α-amylase inhibitory capacity at 62.1% and 24.54%, respectively [103,104]. Some inhibitory peptides and its IC50 and sequences has been detected or identified in literature and are illustrated in Table 2. To our knowledge, reports about inhibitory α-amylase peptides were limited, and less references could be obtained on the α-glucosidase inhibitory peptides in literature.

## 4. The Structure Characteristics of Antidiabetes Peptides

Different approaches have been suggested potential for the treatment and management of Type 2 diabetes by natural peptides. A limited number of studies focus on the structural features that govern the properties of peptides in literature, including the structure of DPP-IV inhibitory, insulinotropic and α-amylase peptides.

For DPP-IV inhibitory peptides, they exert their effect by binding either at the active site and/or outside the catalytic center of the enzyme. In silico studies predicted that the active site of DPP-IV comprises a hydrophobic S1 (Tyr662 and Tyr666) pocket and a charged S2 (Phe357 and Arg125) pocket with an overall negative charge [110,111]. Hydrogen bonds and hydrophobic interactions were involved between N-terminal amino acids of DPP-IV inhibitory peptides and the catalytic active site of DPPIV. Thus, the structural features of DPP-IV inhibitors generally were inferred as a hydrophobic or aromatic amino acid at the N-terminus, such as Ile, Leu, Val, Phe, Trp or Tyr [52]. However, several non-inhibitory peptides possessing hydrophobic or aromatic amino acids at their N-terminus were also found (Table 1). Statistically, 77% of all hydrophobic peptides (with an hydrophobicity index >0) and 53% of the hydrophilic peptides were detected with DPP-IV inhibition [46]. It indicates that N-terminal hydrophobicity or aromaticity is a desirable characteristic, but not sufficient for inhibition.

To investigate the preferential amino acids involving in DPP-IV inhibition, the amount of each amino acid occurring in the DPP-IV inhibitory peptides was calculated based on Table 1. The preferential amino acids were also found as Pro, Leu, Gly, Ala, Trp in decreasing order, and Pro occurred most frequently in DDP-IV inhibitory peptides. Studies also showed that collagen from fish and mammals has also attracted notable attention as a potential source of DPP-IV inhibitory peptides partly due to its high content in Pro residue [51,52]. In addition, peptides with the presence of a Trp at the N-terminus was found as more potent DPP-IV inhibitors with an IC 50 value <200 mM [65]. A positive correlation between the presence of Trp-containing peptides within plant (hemp, pea, rice and soy) protein hydrolysates and their DPP-IV inhibitory properties have been observed [68].

However, containing the most preferring amino acid, i.e., Pro, Ile-Pro and Pro-Tyr were shown to be a DPP-IV inhibitor while Pro-Ile and Tyr-Pro were not [69,112]. The results indicated that the different stereochemistry between them may exert a role in the biological activity of the peptide. Indeed, the existence of exclusion volumes in the S1 pocket of DPP-IV might explain the results, which may restrict the access of bulky amino acids and allow access to smaller residues such as Pro, Ala and Gly [113].

In literature, hydrolysis fractions (<1 kDa) of hard-to-cook bean proteins and whey proteins hydrolysis both showed insulin secretagogue action and improved insulin signaling in adipocytes [36,114]. In vitro studies using pancreatic β-cell lines or primary islet cells displayed significant insulinotropic effects of different amino acid residues, including Ala, Leu, Arg and Gln [115,116]. Without carbohydrates, only ingestion of amino acids (Leu, Arg, Ile, Phe and Ala) or milk-derived peptides also exhibited an increase of insulin secretion [117,118]. Furthermore, studies verified that branched chain amino acids were closely associated with insulinotropic effects [115,116,119,120].

Investigation of whey protein hydrolysis showed that the most potent insulinotropic fractions obtained were hydrophilic, which might be responsible for the activity observed [114]. However, other peptides with hydrophobic characteristics may also contribute to the insulinotropic properties of the whey protein hydrolysate [63]. In addition, the levels of Arg and Phe were found to be associated with an insulinotropic activity [116]. According to the results above, it was inferred that free amino acids and dipeptides would be bioavailable and therefore may reach pancreatic β cells in vivo.

The complex mechanisms of these amino acids exerting their action involve mitochondrial metabolism. During fasting periods, glutamine and alanine are important factors to modulate glucagon release from pancreatic α-cells and subsequently influence insulin secretion from β-cells. On the other hand, high glucose levels raise ATP/ADP ratio in β-cells, and inhibit glutamate oxidation to amplify insulin signals [36].

The structural features of α-amylase peptide inhibitor were investigated by Yu et al. [108]. The amino acids, such as Leu, Pro, Gly and Phe, are frequently found in α-amylase inhibitory activity. Peptides with Pro at the N-terminal of Gly or Phe and C-terminal of Phe or Leu were found owning α-amylase inhibitory activity. The positioning of these amino acids at the N- or C-terminal were believed to be the contributors to α-amylase inhibitory activity of peptides extracted from Pinto bean [121]. Some reports showed that the high molecular weights of amino acids with aromatic ring, such as Arg, Phe, Trp, and Tyr, were crucial for interacting with the active site of human pancreatic α-amylases [104,122]. However, the results on the structural active relationship of α-amylase inhibitor peptides are still limited and not many studies have been conducted. Evenly, the data on the structure features for peptides such as potential α-glucosidase inhibitors have not been found in literature [108].

## 5. Conclusions

Nowadays, the discovery of novel ocean bioactive peptides is one of the most exciting new directions of pharmaceutical science due to their nutritional attributes, large output and uniqueness in terms of diversity, and structural and functional features with respect to peptides isolated from terrestrial plants. The diverse nature of T2DM means that food ingredients, such as natural peptides, will be more suitable for combating it and its associated complications than the synthetic and other drugs with significant side effects in the long term. This review concluded that natural origin peptides derived from several kinds of marine organisms, for instance, macro- and micro-algae, marine sponge, fish skin gelatin, and even tuna cooking juice hydrolysates, besides from milk and beans, showed great potential to regulate glucose metabolism for insulin resistance individuals. *Chlorella vulgaris*, one type of marine microalgae with large biomass and high quality protein accounting for over 60% (Wt), has been reported with significant antidiabetic activities in rare clinical trials. This evidence suggested that valuable antidiabetic activities associated with marine bioactive peptides, especially derived from marine microorganisms and their metabolites might be used in future potentialities in nutraceutical and pharmaceutical industries. Investigation of the structural features of peptides linked with anti-diabetic activity, using bioinformatics combined with molecular biological technology, will be a powerful tool to exploit new peptides from abundant marine proteins. In addition, the performances of research studies using human models or clinical trials are necessary in the future for their further application.

## Figures and Tables

**Table 1:** The precursors, sequences, inhibition capacity (IC 50) of some natural origin peptides with dipeptidyl peptidase IV inhibitory activity in literature with IC 50 < 200 μM.

Food	Precursor Protein	Peptide Sequence	IC 50 (μM)	Reference
Plant Protein	Macroalga *Palmaria palmate* protein	ILAP	43.40	[50]
		LLAP	53.67	
		MAGVDHI	159.37	
Collagen	Halibut skin gelatin	SPGSSGPQGFTG	101.6	[51]
		GPVGPAGNPGANGLN	81.3	
		PPGPTGPRGQPGNIGF	146.7	
	Tilapia skin gelatin	IPGDPGPPGPPGP	65.4	
		LPGERGRPGAPGP	76.8	
		GPKGDRGLPGPPGRDG	89.6	
	Tuna cooking juice hydrolysates	PACGGFWISGRPG	96.4	[52]
		CAYQWQRPVDRIR	78	
		PGVGGPLGPIGPCYE	116.1	
Collagen	Deer skin protein	GPVGXAGPPGK	83.3	[53]
		GPVGPSGPXGK	93.7	
Milk protein	α-Lactalbumin	LKPTPEGDL	45	[54]
		LAHKALCSEKL	165	
		LCSEKLDQ	186	
		TKCEVFRE	166	
	β-Lactalbumin	VAGTWY	174	[55]
		IPAVF	44.7	[56]
	Atlantic salmon collagen/gelatin	GPAE	49.6	[52]
		GPGA	41.9	
	Gouda-type cheese	VPITPTL	110	[57]
		VPITPT	130	
		LPQNIPPL	46	
		VAGTWY	174	
		LPQ	82	
	Whey protein	LAHKALCSEKL	165	[58,59,60,61,62,63]
		WLAHKALCSEKLDQ	141	
		LKPTPEGDL	45 9	
		LKPTPEGDLEIL	57	
		WLAHKALCSEKLDQ	141	
		WR	31.4	
		IPIQY	28.2	
		WCKDDQNPHS	75.0	
		TKCEVFRE	166	
		IPA	49	
		VA3, VL, WL, WI	<170	
		LKPTPEGDLE	42	
		LKALPMH	193	
	Milk protein	WA	92.6	[46,64,65,66]
		WR	37.8	
		WK	40.6	
		LPYPY	108.3	
		WQ	120.3	
		WI	138.7	
		WN	148.5	
		YPYY	194.4	
Milk protein	Milk protein	WN	148.5	[46,64,65,66]
		IP	149.6	
		IPI	3.5	
		IPIQY	35.2	
		FLQP	65.3	
		WV	65.7	
		LPVPQ	48.2	
		IPM	73.9	
		HL	143.2	
		VA	168.2	
		WL	43.6	
		WP	44.5	

**Table 2:** The sequences, inhibition capacity (IC 50) and precursors of natural peptides with α-amylase and α-glucosidase inhibitory activity in literature.

Ingredient	Peptides Sequence	IC 50	Precursors	Reference
α-amylase	PPHMLP	1.97 (mg mL^−1^)	Pinto bean	[103]
	PLPWGAGF	8.96 (mg mL^−1^)		
	PPHMGGP	14.63 (mg mL^−1^)		
	PLPLHMLP	18.45 (mg mL^−1^)		
	LSSLEMGSLGALFVCM	20.56 (mg mL^−1^)		
	FFRSKLLSDGAAAAKGALLPQYW	0.02 (μM)	Cumin seed protein	[104]
	RCMAFLLSDGAAAAQQLLPQYW	0.04 (μM)		
	DPAQPNYPWTAVLVFRH	0.03 (μM)		
	RCMAFLLSDGAAAAQQLLPQYW	0.04 (μM)	Cumin seed protein	[105]
	DPAQPNYPW TAVLVFRH	0.15 (μM)		
	WEVM	-	Black bean protein	[106]
	AKSPLF	-		
	<3 kDa fraction	-	Rice bran protein	[107]
	KLPGF	120.0 ± 4.0 (μM)	Albumin	[108]
	NVLQPS	110.0 ± 6.2 (μM)		
α-glucosidase	-	36.3%–50.1% mg^−1^ DW	*Bean protein*	[109]
	TTGGKGGK	-	Black bean protein	[107]
	KLPGF	59.5 ± 5.7 (μM)	Albumin	[108]
	NVLQPS	100.0 ± 5.7 (μM)		
